# A novel robotic traction system for endoscopic submucosal dissection – achieving en bloc resection of an extensive laterally spreading tumor in the lower rectum

**DOI:** 10.1055/a-2760-9595

**Published:** 2026-01-13

**Authors:** Zhiyong Zhai, Chongju Bao, Wanjun Li, Chao Yang, Wei Gong

**Affiliations:** 1 559569 Department of Gastroenterology, Shenzhen Hospital, Southern Medical University, Shenzhen, China; 2 70570 The Third School of Clinical Medicine, Southern Medical University, Shenzhen, China

**Fig. 1:**
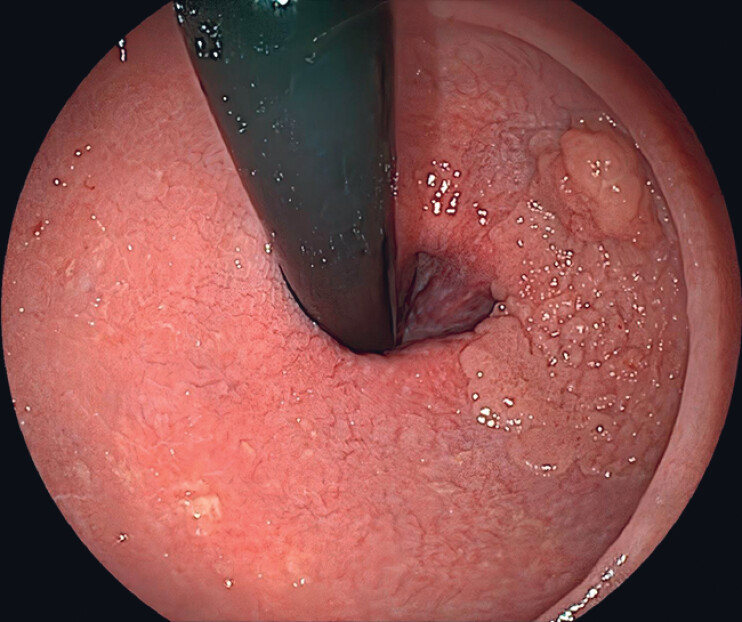
A laterally spreading tumor (5.5 cm × 4.0 cm) with Parisian Type 0-IIa in the lower rectum.

**Fig. 2:**
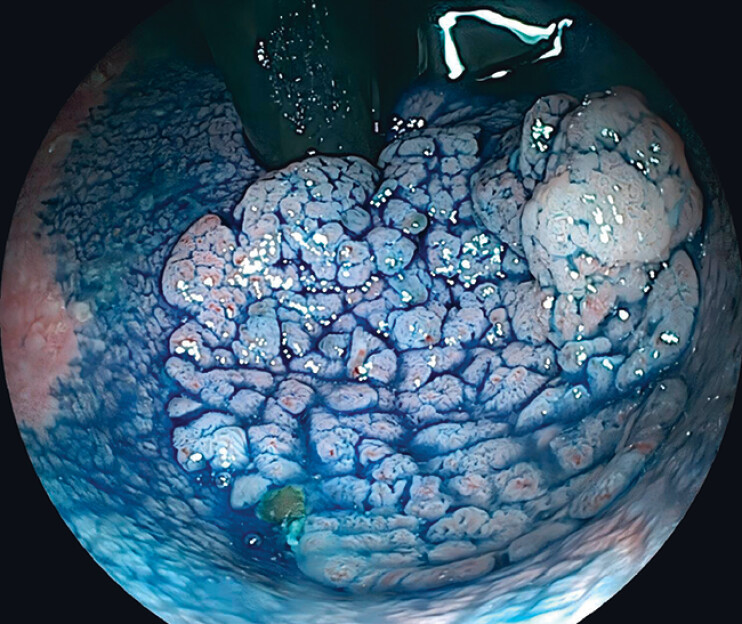
Indigo carmine staining displayed a glandular pattern consistent with Kudo type IV pits.

Colonoscopy revealed a 5.5 × 4.0 cm laterally spreading tumor in the lower rectum near the anal verge, with a well-demarcated line and relatively regular nodules ( Fig. 1 ). Indigo carmine staining displayed a glandular pattern consistent with Kudo type IV pits ( Fig. 2 ), and endoscopic submucosal dissection (ESD) was deemed feasible.

**Fig. 3:**
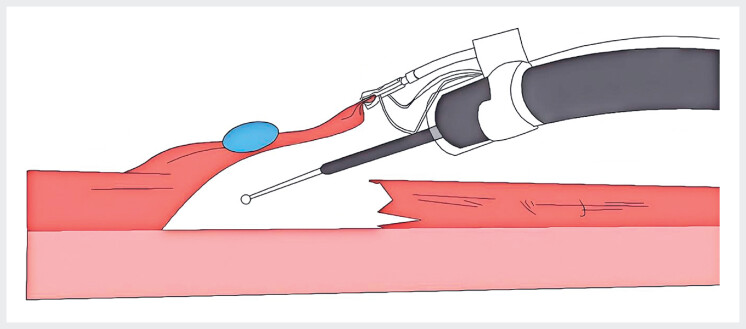
The scheme of the EndoFaster robotic traction system.

**Fig. 4:**
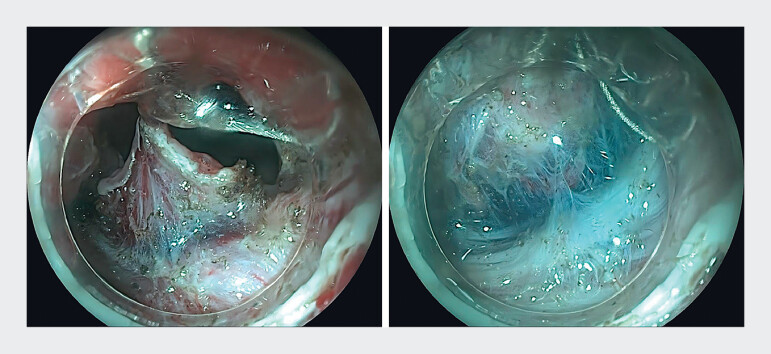
With robot-assisted traction, the submucosal layer was in full exposure (left) and blood vessels were clearly visible (right).

**Fig. 5:**
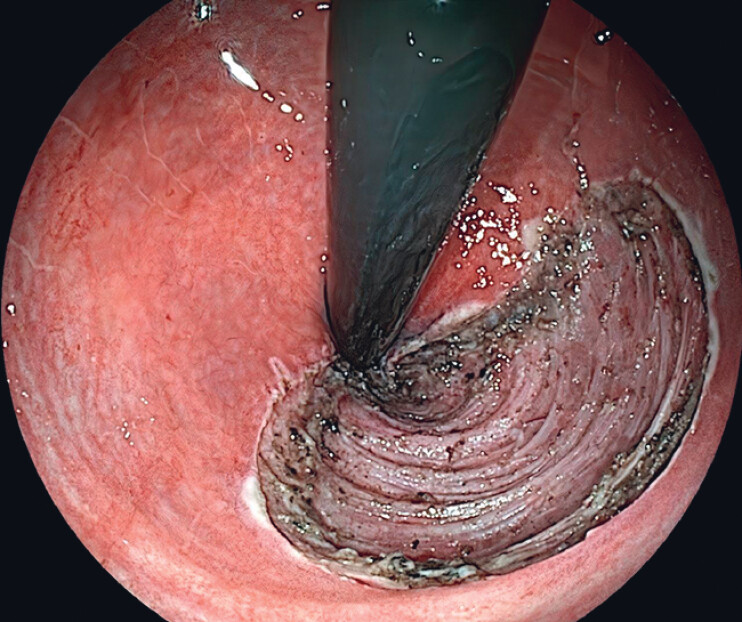
The lesion was successfully completely removed.

Given the lesion’s large size, location in the lower rectum, and rich vascularity, submucosal layer visualization was expected to be suboptimal during dissection, elevating the risks of intraoperative bleeding and incomplete resection. To overcome these challenges, the EndoFaster robotic traction system was employed ( Fig. 3 ). Following submucosal injection and circumferential mucosa incision using a hybrid knife, the device’s white soft hood was attached to the endoscope tip. Grasping forceps were fixed at the 12 o’clock direction to grasp the lesion edge, providing upward traction. The submucosal layer was in full exposure and blood vessels were clearly visible ( Fig. 4 ). The operation progressed with an antegrade approach from the anal to the oral side. By dynamically adjusting the position of the forceps, they can exert a controlled pulling force in multiple directions, which allowed precise electrocoagulation hemostasis and systematic dissection. Finally, the lesion was successfully completely removed ( Fig. 5 ). The total duration of the submucosal dissection was significantly reduced to approximately 30 minutes, with no intraoperative complications ( Video 1 ).

A novel robotic traction system for endoscopic submucosal dissection – achieving en bloc resection of an extensive laterally spreading tumor in the lower rectum.Video 1

Compared with conventional traction methods (e.g. dental floss 1 2 and figure-of-eight clip 3 4 ), EndoFaster enables multiple-position and multiple-angle traction due to its flexibility, which improves dissection efficiency for large lesions in anatomically challenging locations, increases en bloc resection rates, and reduces intraoperative complications, as evidenced by its successful use in a gastric angle lesion by Cui et al. 5 in 2024. Our presented case further exemplifies these advantages and marks its first application in colorectal ESD in China. EndoFaster facilitates ESD procedures, enhancing their safety and thereby offering a novel strategy for colorectal ESD. More cases and a longer follow-up are required to validate the advantages of this technique.

Endoscopy_UCTN_Code_TTT_1AQ_2AD_3AD
